# MCM4 and MCM7, potential novel proliferation markers, significantly correlated with Ki-67, Bmi1, and cyclin E expression in esophageal adenocarcinoma, squamous cell carcinoma, and precancerous lesions✩^,^✩✩

**DOI:** 10.1016/j.humpath.2016.07.013

**Published:** 2016-07-29

**Authors:** Bonnie Choy, Amy LaLonde, Jianwen Que, Tongtong Wu, Zhongren Zhou

**Affiliations:** a Department of Pathology, University of Chicago, Chicago, IL 60637; b Department of Biostatistics and Computational Biology, University of Rochester Medical Center, Rochester, NY 14642; c Center for Human Development & Division of Digestive and Liver Diseases, Columbia University Medical Center, NY 10032; d Department of Pathology and Laboratory Medicine, University of Rochester, Rochester, NY 14642

**Keywords:** MCM4, MCM7, Ki-67, Esophageal adenocarcinoma, Esophageal squamous cell carcinoma, Barrett's esophagus

## Abstract

Minichromosomal maintenance (MCM) proteins are participants of DNA replication and may represent more accurate markers in determining the proliferative fraction within a tumor than proliferative marker Ki-67. Our study investigated the correlation between MCM4 and MCM7 expression and Ki-67, Bmi1, and cyclin E expression in esophageal adenocarcinoma, squamous cell carcinoma, and precancerous lesions. MCM4 and MCM7 expression had similar distribution as Ki-67 and Bmi1 expression in esophageal carcinoma and pre-cancerous lesions. The mean percentage of MCM4, MCM7, and Ki-67 expression increased from squamous epithelium (5.5%, 7.3%, and 5.9%, respectively), to columnar cell metaplasia (11.2, 13.5%, and 3.4%), Barrett's esophagus (27.7%, 35.3%, and 8.3%), low-grade dysplasia (42.6%, 52.2%, and 12.9%), high-grade dysplasia (63.2%, 77.7%, and 29.6%), adenocarcinoma (61.3%, 75.5%, and 24.5%), and squamous cell carcinoma (74.1, 85.4%, and 36.3%). The percentages of MCM4 and MCM7 expression were significantly higher than Ki-67 expression. Using univariate analysis we found a high percentage of MCM4 expression (>70%) to be significantly associated with lymph node metastasis and shorter survival in the adenocarcinoma group. We also demonstrated the percentage of MCM4 and MCM7 expression to be significantly correlated with Ki-67, Bmi1, and cyclin E expression in esophageal carcinoma and precancerous lesions. MCM4 and MCM7 may serve as more sensitive proliferative markers for the evaluation of esophageal lesions.

## 1. Introduction

The minichromosomal maintenance (MCM) protein family consists of six related proteins that have essential roles in the initiation of DNA replication [1]. MCM proteins are also involved in the elongation of DNA replication and other chromosome transactions including damage response, transcription, and chromatin structure [2,3]. Deregulation of the MCM proteins contributes to cell proliferation and tumorigenesis. Aberrant expressions of MCM proteins have been reported to be promising prognostic markers in a number of malignancies [4-17].

It has been claimed that MCM proteins are potentially more accurate in determining the proliferative fraction within a tumor than conventional proliferative markers such as Ki-67 [10]. The presence of MCM2, MCM5, and Ki-67 expression was previously reported in esophageal squamous dysplasia and Barrett's esophagus with glandular dysplasia [18,19]. Further, studies have observed MCM2 expression in esophageal squamous cell carcinoma and its positive correlation with Ki-67 expression [18,20]. MCM4 mRNA expression has also been observed in esophageal squamous cell carcinoma [21]. While limited studies on MCM4 and MCM7 expression in esophageal carcinoma have been reported, none of the studies to the best of our knowledge investigated MCM4 and MCM7 expression by immunohistochemistry.

Bmi1 is a member of the polycomb-group proteins and functions as a stem cell marker to regulate the proliferation of progenitor cells [22]. Our previous study demonstrated that Bmi1 expression was similar to Ki-67 expression in their distribution in the basal layer of normal squamous epithelium and extending to full thickness in esophageal carcinoma [23]. Cyclin E plays an important role in promoting G1 cell cycle transition to Sphase [24]. MCM7 has been reported to be the substrate of cyclin E/Cdk2 [25], and high level of MCM4 expression has been associated with cyclin E expression in non–small cell lung carcinoma [10]. In addition, we previously found aberrant expression and amplification of cyclin E significantly increased in dysplastic esophageal lesions [26].

In the current study, we first examined the immunohistochemical expression of MCM4 and MCM7 in comparison to the conventional proliferation marker Ki-67 in esophageal adenocarcinoma, squamous cell carcinoma, and precancerous lesions to determine the predictive value of these biomarkers for the progression of esophageal diseases. Next, we investigated the clinicopathologic association of MCM4, MCM7, and Ki-67 expression in esophageal adenocarcinoma, squamous cell carcinoma, and precancerous lesions. We also explored the correlation between MCM and Bmi1 as well as cyclin E expression.

## 2. Materials and methods

### 2.1. Construction of tissue microarrays

Tissue microarrays were constructed from representative areas of formalin-fixed specimens collected from 1997 to 2005 in the Department of Pathology and Laboratory Medicine, University of Rochester Medical Center, Rochester, NY. The tissue microarrays contained 82 squamous epithelium, 60 columnar cell metaplasia, 33 Barrett's esophagus, 38 low-grade dysplasia, 14 high-grade dysplasia, 108 esophageal adenocarcinoma, and 24 esophageal squamous cell carcinoma. Clinicopathologic data of the patients, including age, gender, TNM stage, histologic grade, and duration of survival, were obtained from the medical records. All patients' identifiers were removed. The study was approved by the institutional review board (Biomarkers of esophageal carcinoma, RSRB28546).

### 2.2. Immunohistochemistry

Immunohistochemical studies were performed on 5-μm thick sections of tissue microarrays. Briefly, after endogenous peroxidase activity was quenched and nonspecific binding was blocked, ready-to-use mouse monoclonal antibodies to MCM4 (1:50; Santa Cruz Biotechnology, Santa Cruz, CA) and MCM7 (1:50; Santa Cruz Biotechnology) were incubated at 4°C overnight, and Ki-67 (1:100; Dako, Carpinteria, CA) was incubated at room temperature for 30 minutes. The secondary antibody (Flex HRP, Dako) was incubated for 30 minutes. After washing, sections were incubated with Flex DAB chromogen for 10 minutes and counterstained with Flex hematoxylin for 5 minutes. Appropriate positive and negative controls were evaluated. Tissue microarrays were also stained with hematoxylin and eosin to be used for histologic comparison. The percentage of positive nuclear expression for MCM4, MCM7, and Ki-67 was reviewed by two pathologists. Various cut-offs were tested to establish high and low expression levels. The percentages close to mean expression levels of MCM4 (70%), MCM7 (70%) and Ki-67 (25%) expression correlated best with overall survival in esophageal carcinoma. The cut-offs were set at 70% for MCM4 and MCM7 and at 25% for Ki-67.

Bmi1 and cyclin E immunostaining were performed as previously described [23,26]. Mouse monoclonal antibodies to Bmi1 (1:100; Millipore, Bedford, MA) and cyclin E (1:100; Santa Cruz Biotechnology) were used for immunohistochemical studies.

### 2.3. Statistical analysis

Pairwise mean comparisons were used to analyze the percentages of immunostaining between the histologic groups: 1) adenocarcinoma, high-grade dysplasia, low-grade dysplasia, Barrett's esophagus, and columnar cell metaplasia, and squamous epithelium; 2) squamous cell carcinoma and squamous epithelium. Pearson's χ^2^ tests, *t* tests, and Fisher exact tests were used as appropriate to assess the association between clinicopathologic characteristics and MCM4, MCM7, and Ki-67 expression. Univariate and multivariate regression models were generated. Probabilities of survival were estimated using the Kaplan–Meier method and were analyzed by log-rank test. All statistical tests were 2-sided. A *P* < .05 was considered to be statistically significant. Statistical analyses were performed using SAS version 9.3 (SAS Institute, Inc, Cary, NC).

## 3. Results

### 3.1. Expression and distribution of MCM4 and MCM7

**Fig 1:**
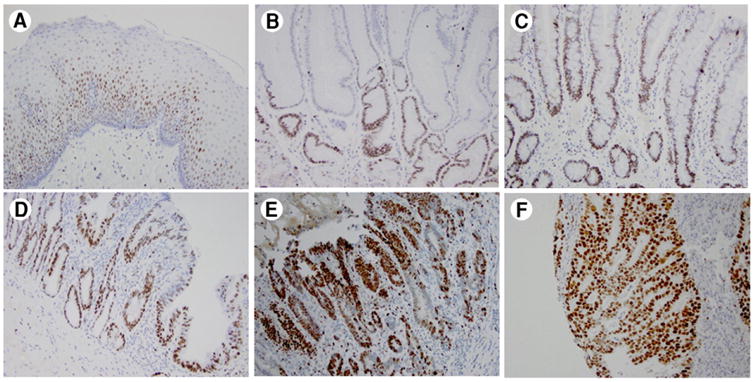
Immunohistochemical analysis of MCM4 in esophageal adenocarcinoma and precancerous lesions. A, Squamous mucosa. B, Columnar cell metaplasia. C, Barrett's esophagus. D, Low-grade dysplasia. E, High-grade dysplasia. F, Esophageal adenocarcinoma. In normal mucosa and non-dysplastic lesions, MCM4 nuclear staining is distributed in the basal layer of the epithelium and lower part of the glands. In dysplastic and cancerous lesions, the glands have full thickness staining for MCM4.

**Fig 2:**
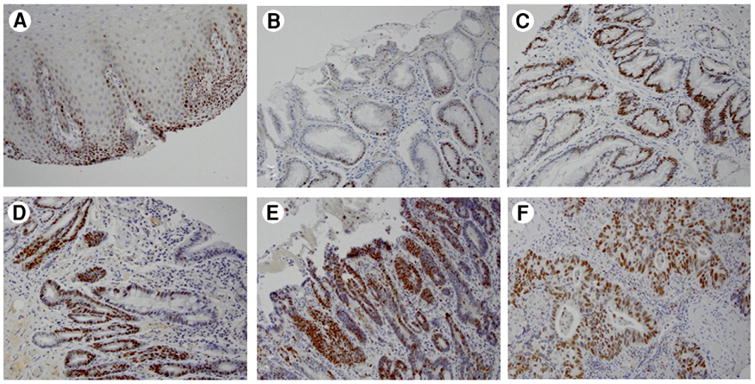
Immunohistochemical analysis of MCM7 in esophageal adenocarcinoma and precancerous lesions. MCM7 is nuclear stain. A, Squamous mucosa. B, Columnar cell metaplasia. C, Barrett's esophagus. D, Low-grade dysplasia. E, High-grade dysplasia. F, Esophageal adenocarcinoma. In normal mucosa and non-dysplastic lesions, MCM7 nuclear staining is distributed in the basal layer of the epithelium and lower part of the glands. In dysplastic and cancerous lesions, the glands have full thickness staining for MCM7.

**Fig 3:**
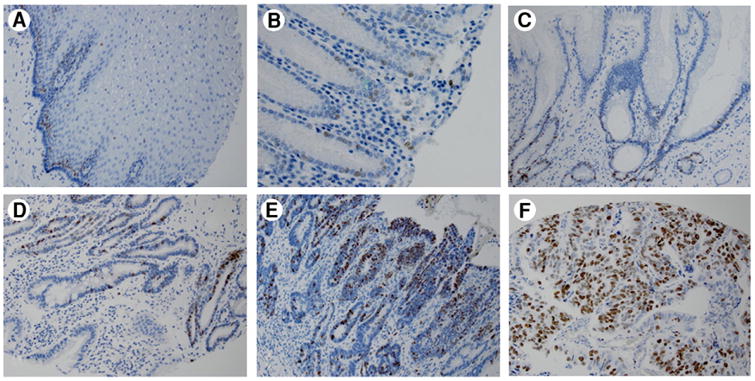
Immunohistochemical analysis of Ki-67 in esophageal adenocarcinoma and precancerous lesions. A, Squamous mucosa. B, Columnar cell metaplasia. C, Barrett's esophagus. D, Low-grade dysplasia. E, High-grade dysplasia. F, Esophageal adenocarcinoma. In normal mucosa and non-dysplastic lesions, Ki-67 nuclear staining is distributed in the basal layer of the epithelium and lower part of the glands. In dysplastic and cancerous lesions, the glands have full thickness staining for Ki-67.

**Fig 4:**
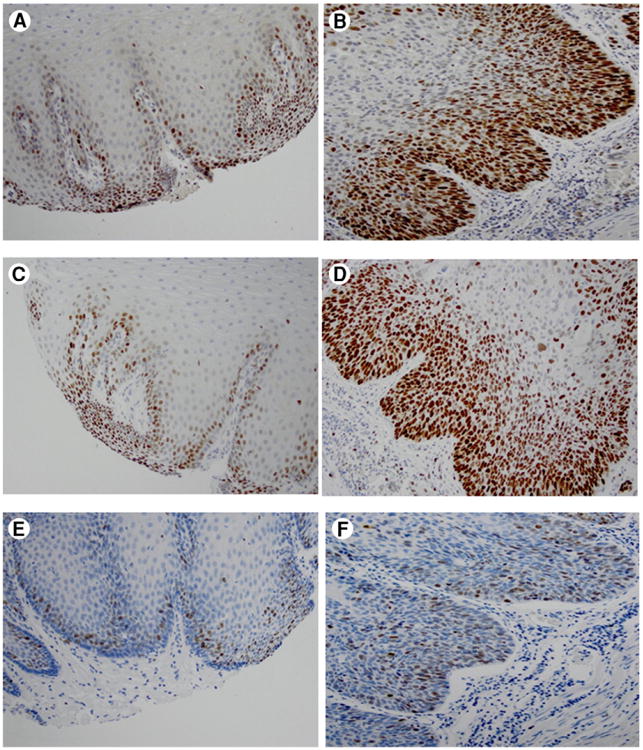
Immunohistochemical analysis of MCM4, MCM7, and Ki-67 in esophageal squamous cell carcinoma. A, MCM4 in squamous mucosa. B, MCM4 in esophageal squamous cell carcinoma. C, MCM7 in squamous mucosa. D, MCM7 in esophageal squamous cell carcinoma. E, Ki-67 in squamous mucosa. F, Ki-67 in esophageal squamous cell carcinoma.

In squamous epithelium, MCM4 and MCM7 expression were scattered in the basal layer, but more diffusely in the parabasal or suprabasal layers. While MCM4 and MCM7 expression were located mainly at the base of glands in columnar cell metaplasia and Barrett's esophagus, their expression extended superficially to involve entire glands as the lesions progressed from dysplasia to adenocarcinoma (Figs. 1 and 2). In addition, MCM4 and MCM7 expression showed more reactivity on the surface of glands compared with the base of glands in high-grade dysplasia. These distributions of immunostaining were comparable to those of Ki-67 (Fig. 3). All three immunomarkers demonstrated full-thickness staining in squamous cell carcinoma (Fig. 4).

The mean percentages of MCM4 and MCM7 expression increased from squamous epithelium (6% and 7%) to columnar cell metaplasia (11% and 14%) and Barrett's esophagus (28% and 35%). In low-grade dysplasia, the mean percentages increased to 43% and 52%, respectively. The mean percentages further increased to 63% and 78% in high-grade dysplasia and 61% and 76% in adenocarcinoma. The mean percentages of MCM4 and MCM7 expression were also high in squamous cell carcinoma (74% and 85%). For Ki-67, the mean percentages of expression were 6% in squamous epithelium, 3% in columnar cell metaplasia, 8% in Barrett's esophagus, 13% in low-grade dysplasia, 30% in high-grade dysplasia, 25% in adenocarcinoma, and 36% in squamous cell carcinoma. The mean percentages of MCM4 and MCM7 expression are significantly higher than that of Ki-67 expression in all categories except for squamous epithelium.

Pairwise mean comparisons found the percentages of MCM4, MCM7, and Ki-67 expression in esophageal adenocarcinoma and high-grade dysplasia to be significantly greater than those in low-grade dysplasia, Barrett's esophagus, columnar cell metaplasia, and squamous epithelium (*P* < .05) (Table 1). There were also significant differences when comparing the percentages of MCM4 and MCM7 expression in (1) low-grade dysplasia with Barrett's esophagus, columnar cell metaplasia, and squamous epithelium, and (2) Barrett's esophagus with columnar cell metaplasia and squamous epithelium. Additionally, the percentages of MCM4, MCM7, and Ki-67 expression in squamous cell carcinoma were significantly greater than those in squamous epithelium.

### 3.2. Correlations between MCM4, MCM7 and Ki-67 and clinicopathologic features

Correlations between MCM4, MCM7, and Ki-67 expression and patients' clinicopathologic characteristics, including age, gender, TNM stage, and histologic grade, were analyzed in the adenocarcinoma group (Table 2) and the squamous cell carcinoma group (Table 3). Only univariate analysis identified a significant association between MCM4 expression and lymph node metastasis in the adenocarcinoma group. However, multivariate analysis did not yield any significant association.

### 3.3. Association of MCM4 and MCM7 with overall survival

**Fig 5:**
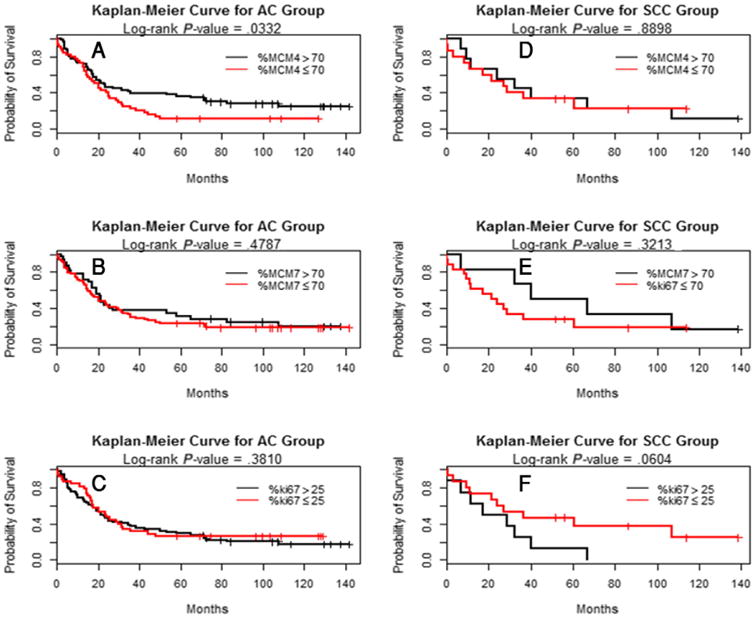
Kaplan-Meier survival curves for MCM4, MCM7, and Ki-67 in the esophageal adenocarcinoma group (A, B, and C, respectively) and squamous cell carcinoma group (D, E, and F, respectively). The overall survival time for patients with high percentage of MCM4 expression (>70%) was statistically shorter than patients with low percentage (≤70%) in the esophageal adenocarcinoma group (*P* = .03; A), but not in the squamous cell carcinoma group (D). No statistical significance was found in MCM7 and Ki-67 expression in both esophageal adenocarcinoma (B and C) and squamous cell carcinoma (E and F).

Various cut-offs between high- and low-level expression were tested. Values close to mean percentages of MCM4 (70%), MCM7 (70%) and Ki-67 (25%) expression had the best association between MCM4, MCM7 and Ki-67 expression and overall survival in esophageal carcinoma. Kaplan– Meier survival curves to analyze the difference between high and low expression level for MCM4, MCM7, and Ki-67 were generated based on overall survival in patients with esophageal adenocarcinoma and those with squamous cell carcinoma (Fig. 5). In the adenocarcinoma group, the overall survival time for patients with high MCM4 expression level (mean, 27.1 months) was statistically shorter than those with low MCM4 expression level (mean, 46.5 months) (*P* = .03). However, the overall survival time between high (mean, 34.8 months) and low (mean, 48.5 months) expression levels for MCM4 lacked significance in the squamous cell carcinoma group. For MCM7, neither high (mean, 35.8 months) nor low (mean, 43.9 months) expression levels in the adenocarcinoma group and neither high (mean, 31.5 months) nor low (mean, 65.2 months) expression levels in the squamous cell carcinoma group demonstrated significant difference in overall survival. Similar findings were observed for Ki-67 with high (mean, 39.9 months) and low levels of expression (mean, 37.6 months) in the adenocarcinoma group and high (mean, 49.7 months) and low levels of expression (mean, 23.6 months) in the squamous cell carcinoma group.

### 3.4. Correlation of MCM4 and MCM7 with Ki-67, Bmi1 and cyclin E

All correlations between MCM4, MCM7, and Ki-67 expression, as well as with Bmi1 and cyclin E were positive and significant. All correlation values between MCM4, MCM7, and Ki-67 expression were greater than 0.65 and *P* < .0001.

## 4. Discussion

The MCM protein family is involved in a number of essential steps in DNA replication [1]. Their roles in DNA replication make them candidates as proliferation markers [8]. In our current study, we compared two members of the MCM protein family, MCM4 and MCM7, to the conventional proliferation marker Ki-67, stem cell marker Bmi1, and cell cycle promoter cyclin E.

The literature on MCM4 and MCM7 expression in esophageal cancer is limited. Via reverse-transcription polymerase chain reaction (RT-PCR), MCM4 expression was reported to be increased in esophageal squamous cell carcinoma when compared with normal epithelium. MCM4 expression was also increased in stage T3 carcinoma when compared with stage T1 carcinoma [27]. By microRNA microarrays and quantitative RT-PCR, MCM7 mRNA expression and DNA copy number at the MCM7 locus were found to be up-regulated and increased in esophageal adenocarcinoma with disease progression [28]. In cervical cancer, bladder cancer, cutaneous melanoma, and oral squamous cell carcinoma, MCM4 and MCM7 have been reported to be promising prognostic markers for disease progression [4,5,12,14,16]. This is the first time immunohistochemistry is used to demonstrate that the percentages of MCM4 and MCM7 expression significantly increased with disease progression and strongly correlated with Ki-67 expression.

MCM4 and MCM7 expression were scattered in the basal layer where the stem-like cells are located [29], but more diffusely in the parabasal or suprabasal layers in squamous epithelium. While MCM4 and MCM7 expression were located mainly at the base of glands in columnar cell metaplasia and Barrett's esophagus, their expression extended superficially to involve entire glands as the lesions progressed from dysplasia to adenocarcinoma (Figs. 1 and 2). Similar findings were reported in previous studies [18,19]. Two studies on MCM2 and MCM5 in the upper gastrointestinal tract found no expression on the luminal surface of normal squamous esophagus, gastric antrum, and duodenum. In addition, MCM2 and MCM5 expression were observed to gradually extend towards the surface and upper portion of crypts with increasing degree of dysplasia [18,19]. Our studies further confirmed that MCM4 and MCM7 expression had similar distribution as conventional proliferation marker Ki-67 and had significant correlation with Ki-67. These findings suggest that MCM proteins are potential proliferation markers. In pairwise mean comparisons, the percentages of MCM4 and MCM7 expression was significantly greater than the percentages of Ki-67 expression in esophageal carcinoma and most of the precancerous lesions. It has been claimed that MCM proteins are more accurate means of determining the proliferative fraction within a tumor than conventional proliferation markers, such as Ki-67, because the latter fails to label cells in the early G1 phase or is down-regulated early in the differentiation program [10]. The significant increase of MCM4 and MCM7 expression compared with Ki-67 suggests that MCM4 and MCM7 are potentially more sensitive markers in differentiating various stages of esophageal disease progression.

Pairwise mean comparisons found the percentages of MCM4 and MCM7 expression significantly increased from columnar cell metaplasia, Barrett's esophagus, low-grade dysplasia to high-grade dysplasia (*P* < .05), except for squamous mucosa versus columnar cell metaplasia and esophageal adenocarcinoma versus high-grade dysplasia (Table 1). In addition, the distribution of MCM4- and MCM7-positive cells gradually extended from the basal layer to entire glands(Figs. 1 and 2). Our findings suggest MCM4 and MCM7 proteins are potentially helpful as proliferation markers in the diagnosis of challenging cases of dysplasia. However, additional studies are needed to identify the specific cut-offs of MCM4 and MCM7 expression and specific distribution to differentiate dysplasia from reactive changes, which is beyond the scope of the current study.

In a number of malignancies, aberrant expression of MCM proteins has been associated with poorer prognosis [5-7,13,17,18,30]. MCM4 was reported to be a promising marker for distinguishing benign from malignant melanocytic skin lesions and to be associated with shorter survival in patients with melanoma [30]. In breast cancer, high level of MCM4 expression was associated with disease progression, ER-negative or high-grade breast tumors, and shorter survival [11]. In the gastrointestinal tract, MCM7 expression was found to be a poor prognostic factor for gastric and colorectal cancer [31,32]. Studies also showed MCM7 expression had poorer prognosis in lung adenocarcinoma [13,33]. However, the association between MCM expression and prognosis remains controversial in other malignancies. One study found that MCM7 expression was associated with better prognosis in serous carcinoma of the ovary [15]. Another study reported no association between MCM4 expression and survival in non–small cell lung carcinoma [10]. Our analysis demonstrated that patients with high MCM4 expression level (>70%) had significantly shorter survival in the adenocarcinoma group. However, no significant difference in survival time was found in the squamous cell carcinoma group. MCM7 and Ki-67 expression also showed no significant difference in survival time in both the adenocarcinoma and squamous cell carcinoma groups.

A previous study of MCM4 expression in esophageal squamous cell carcinoma found a significant association between increased expression and higher histologic stage [27]. We did not find an association between MCM expression and clinicopathologic characteristics. Small sample number in the squamous cell carcinoma group may be a potential reason. The only significant association observed was between MCM4 expression and lymph node metastasis on univariate analysis. This association may be related to significantly shorter survival in the esophageal adenocarcinoma group with high MCM4 expression level (>70%).

MCM4 and MCM7 expression strongly correlated with Bmi1 and cyclin E expression. Bmi1 is a stem cell marker with similar distribution to those of MCM4, MCM7, and Ki-67 in esophageal adenocarcinoma and precancerous lesions [23]. The similar distribution suggests MCM proteins may play an important role in the proliferation and/or DNA replication of early progenitor cells [1,28]. Further studies are needed to explore the underlying mechanism between MCM and Bmi1. High MCM4 expression level was reported to correlate with cyclin E expression in non–small cell lung carcinoma by DNA replication [10]. In addition, budding yeast MCM4 is phosphorylated in vivo during S phase in a manner dependent on the presence of five CDK phosphoacceptor residues to trigger DNA replication [34]. Our study further confirmed that MCM4 expression correlated with cyclin E expression in esophageal carcinoma. In addition, MCM7 was reported to be a substrate of cyclin E/*CDK2* and can be phosphorylated on Ser-121 [25]. It has been suggested that phosphorylation of MCM7 on Ser-121 is involved in preventing DNA replication, as well as in the regulation of mitotic exit. Our results showed that MCM7 expression was strongly associated with cyclin E expression, suggesting that MCM7 may be involved in the esophageal cell cycle as a substrate of cyclin E/*CDK2*. In summary, our findings demonstrated the percentages of MCM4 and MCM7 expression significantly correlated with Ki-67, Bmi1, and cyclin E expression in esophageal carcinoma and precancerous lesions. MCM4 and MCM7 may serve as more sensitive proliferation markers for evaluation of esophageal carcinoma and precancerous lesions. Higher percentage of MCM4 expression also showed significantly worse prognosis in patients with esophageal adenocarcinoma and is associated with lymph node metaplasia, making MCM4 a better proliferative marker.

## Figures and Tables

**Table 1:** Pairwise mean comparisons of percentages of MCM4, MCM7, and Ki-67 expression between esophageal histologic types

	Comparison	Difference in percentage of expression		

		MCM4	MCM7	Ki-67
Adenocarcinoma	High-grade dysplasia	−1.9	−2.2	−16.8
	Low-grade dysplasia	18.8 **	23.3 **	11.6 **
	Barrett's esophagus	33.7 **	40.2 **	16.2 **
	Columnar cell metaplasia	50.2 **	62 **	26.3 **
	Squamous epithelium	55.9 **	68.1	18.6
High-grade dysplasia	Low-grade dysplasia	20.6 **	25.5 **	16.8 **
	Barrett's esophagus	35.5 **	42.4 **	21.3 **
	Columnar cell metaplasia	52 **	64.2 **	26.3 **
	Squamous epithelium	57.8 **	70.4 **	23.7 **
Low-grade dysplasia	Barrett's esophagus	14.9 **	16.9 **	4.6
	Columnar cell metaplasia	31.4 **	38.7 **	9.5
	Squamous epithelium	37.1 **	44.9 **	7 **
Barrett's esophagus	Columnar cell metaplasia	16.5 **	21.8 **	4.9
	Squamous epithelium	22.2 **	28 **	2.4
Columnar cell metaplasia	Squamous epithelium	5.7	6.2	−2.5
Squamous cell carcinoma	Squamous epithelium	68.6 **	78.1 **	30.4 **

** *P* < .05.

**Table 2:** Clinicopathologic characteristics and percentages of MCM4, MCM7, and Ki-67 expression for esophageal adenocarcinoma group

	MCM4 (mean %; SD)	*P*	MCM7 (mean %; SD)	*P*	Ki-67 (mean %; SD)	*P*
Gender						
Male	61.6 (26.6) n = 97	.71	75.9 (23.5) n = 94	.81	25 (23) n = 90	.5
Female	59.1 (26.2) n = 11		71.8 (27.5) n = 11		20 (22.5) n = 10	
TNM staging						
T						
1	55 (21.2) n=2	.6	60 (28.3) n=2		7.5 (3.5) n=2	.36
2	58 (24.3) n = 15		75 (25.1) n = 15		19.7 (21.8) n = 15	
3	56.1 (30.7) n = 23		72.9 (25.3) n = 21	.64	21.8 (21.7) n = 20	
4	64 (25.7) n = 68		76.9 (23.4) n = 67		27.1 (23.7) n = 63	
N						
0	50 (27.7) n = 28	.04 **	68 (27.2) n = 28	.09	15 (13) n = 27	.07
1	67.8 (24.1) n = 54		81.3 (19.9) n = 53		30.1 (25.5) n = 49	
2	63.3 (21) n = 15		72.9 (19) n = 14		21.7 (23.8) n = 15	
3	55.7 (33.2) n = 11		69 (33.5) n = 10		27.2 (22.7) n=9	
Histological grade						
Poor	61.4 (26.3) n = 67	.7	73.8 (24.1) n = 64	.35	24 (22.3) n = 63	.81
Moderate	61.7 (26.8) n = 33		78.2 (24.4) n = 33		25.3 (24.7) n = 30	
Well	51.8 (30.6) n=6		71.7 (21.4) n=6		17 (15.7) n=5	

** *P* < .05.

**Table 3:** Clinicopathologic characteristics and percentages of MCM4, MCM7, and Ki-67 expression for esophageal squamous cell carcinoma group

	MCM4 (mean %;SD)	*P*	MCM7 (mean %;SD)	*P*-value	Ki-67 (mean %;SD)	*P*
Gender						
Male	74.8 (23.5) n = 14	.84	86.4 (19.8) n = 14	.59	36.2 (24.6) n = 13	.93
Female	73 (22.8) n = 10		84 (19.6) n = 10		36.5 (29.3) n = 10	
TNM staging						
T						
1	95 (N/A) n=1	.14	100 (N/A) n=1	.29	60 (N/A) n=1	.71
2	49.2 (37.4) n=3		66.7 (30.6) n=3		31.7 (27.5) n=3	
3	50 (N/A) n=1		70 (N/A) n=1		20 (N/A) n=1	
4	78.2 (18) n = 19		88.4 (16.8) n = 19		36.7 (27.2) n = 18	
N						
0	72.8 (34.4) n=8	.68	83.8 (27.2) n=8	.82	33.8 (27.1) n=8	.28
1	72.7 (13.5) n = 11		84.5 (15.7) n = 11		41.5 (27.7) n = 10	
2	81.7 (12.6) n=3		93.3 (11.5) n=3		46.7 (11.5) n=3	
3	75 (35.4) n=2		85 (21.2) n=2		5 (7.1) n=2	
Histological grade						
Poor	68.3 (26) n = 10	.35	82 (20.4) n = 10	.49	32.2 (29.4) n=9	.64
Moderate	76.9 (20.2) n = 13		86.9 (19.3) n = 13		37.3 (24.7) n = 13	
Well	N/A		N/A		N/A	

Abbreviation: N/A, not applicable.
